# Occurrence and molecular characterization of *Cryptosporidium* in dogs in Henan Province, China

**DOI:** 10.1186/1746-6148-10-26

**Published:** 2014-01-17

**Authors:** Fuchun Jian, Meng Qi, Xiaoyi He, Rongjun Wang, Sumei Zhang, Heping Dong, Longxian Zhang

**Affiliations:** 1 College of Animal Science and Veterinary Medicine, Henan Agricultural University, Zhengzhou 450002, P R, China; 2 International Joint Research Laboratory for Zoonotic Diseases of Henan, Zhengzhou 450002, P R, China; 3 Xiangya School of Medicine, Central South University, Changsha 410013, P R, China

**Keywords:** Infection rate, Dogs, *Cryptosporidium canis*, SSU rRNA, HSP70, Actin

## Abstract

**Background:**

Cryptosporidiosis in dogs has been reported worldwide, involving both asymptomatic and diarrheic dogs. Large-scale surveys of *Cryptosporidium* infection in dogs have been performed in some countries using differents diagnostic methods. But, few data are available on the infection rate and molecular characteristics of *Cryptosporidium* spp. in dogs in China.

**Result:**

In this study, 770 fecal samples from 66 locations in Henan Province were examined. The average *Cryptosporidium* infection rate was 3.8%, with dogs in kennels having the highest rate of 7.0% (*χ*^2^ = 14.82, *P* < 0.01). The infection rate was 8.0% in dogs younger than 90 days, which was significantly higher than that in the other age groups (1.1–3.8%;*χ*^2^ = 18.82, *P* < 0.01). No association was noted between the infection rate and the sex of the dogs. Twenty-nine *Cryptosporidium*-positive samples were amplified at the small subunit rRNA (SSU rRNA), 70-kDa heat shock protein (HSP70), and actin loci using PCR. Sequence analysis of these amplicons identified only *Cryptosporidium canis*, which showed 100% identity with the published sequences of the SSU rRNA, HSP70, and actin genes.

**Conclusions:**

Our results confirm that *C. canis* is popular in the dog population in China, considering the large number of dogs in China and the close contact between dogs and humans, the role of *C. canis* in the transmission of human cryptosporidiosis warrants attention.

## Background

*Cryptosporidium* spp. are important zoonotic protozoan parasites that infect more than 280 species of vertebrate animals and can cause acute or chronic diarrhea and even death [1]. So far, at least 26 valid *Cryptosporidium* species have been named, and more than 73 host-adapted genotypes of undetermined species status have been described [1,2].

Dogs are often considered to be faithful friends and intimate companions of humans. However, dogs and cats also act as reservoirs for a large number of pathogens of parasitic zoonoses, including cryptosporidiosis. *Cryptosporidium canis*, the most frequently identified species of *Cryptosporidium* in dogs, has been found in stray and domesticated dogs worldwide [3-10], although small numbers of zoonotic *C. parvum*, *C. muris*, and *C. meleagridis* have also been detected in dogs [7,11-16]. *Cryptosporidium canis* is considered a potentially zoonotic *Cryptosporidium* species based on its presence in human patients in the United Kingdom, Jamaica, Kenya, Peru, Thailand, and the United States [1,17,18].

In China, the history of raising dogs goes back more than 6000 years [19]. The total number of dogs in China at present is vast, with the number of pet dogs alone approaching 200 million [20]. This large number of dogs may be a serious threat to public health because of the widespread occurrence of rabies and other zoonotic diseases, including cryptosporidiosis. Unfortunately, only a few studies of cryptosporidiosis in dogs in China have been reported, all of which were published in the Chinese language and none of which included genotype analyses [21-23]. Therefore, in this study, we used molecular methods to identify the *Cryptosporidium* species present in a relatively extensive survey of dogs and evaluated the risk of zoonotic infections.

## Results and discussion

The overall infection rate with *Cryptosporidium* was 3.8% (29/770). This is similar to the rates in studies conducted in the United States (2.0 and 3.8%) and Italy (3.3%), and one survey in China (2.59%) [6,28,29], whereas the infection rate was slightly lower than those reported in Japan (3.9%), Iran (5%), and Canada (9.3%) [9,10,14]. No clinical signs were apparent in any of the *Cryptosporidium*-positive dogs at the time of specimen collection. The infection rates of *Cryptosporidium* spp. in dogs in kennels, pet shops, pet hospitals, and the police canine center were 7.0% (16/229; 95% CI: 7.0 ± 0.23%), 4.5% (11/246; 95% CI: 4.5 ± 0.17%), 1.0% (2/206; 95% CI: 1.0 ± 0.09%), and 0 (0/89), respectively (Table 1). These differences might be partly attributable to the various feeding and management practices used and to hygienic conditions. For example, in the police canine center, each dog was kept in a single shed and the feces excreted by the dogs were cleared in a timely manner by the dogs’ handlers, whereas the dogs in kennels were kept together, which increased the chance of cross-transmission among them.

**Table 1:** **Number of fecal samples from different sampling locations examined for *Cryptosporidium *** **oocysts with microscopy**

**Collection site**	**No. samples**	**No. positive**	**Prevalence (95% CI) (%)**
Kennel	229	16	7.0 ± 0.23
Pet shop	246	11	4.5 ± 0.17
Pet hospital	206	2	1.0 ± 0.09
Police canine center	89	0	0
Total	770	29	3.8 ± 0.05

The *Cryptosporidium* infection rates differed with age. The highest infection rate was 8.0% (95% CI: 8.0 ± 0.06%) and was found among dogs aged < 90 days and the lowest rate of 1.1% (95% CI: 1.1 ± 0.08%) was found among dogs aged > 360 days (*χ*^2^ = 18.82, *P* < 0.01; Table 2). The decreasing *Cryptosporidium* infection rate with increasing age is consistent with several recent studies conducted in Norway and China [28,30,31]. In contrast, no significant relationship was seen between the occurrence of *Cryptosporidium* and the sex of the dogs: the infection rate was 4.2% (15/353) in female dogs and 3.4% (14/417) in male dogs (*χ*^2^ = 0.42, *P* > 0.05). Two studies conducted in China and Brazil reported similar findings [22,32]. This indicates that sex is not a major issue affecting *Cryptosporidium* infection in dogs.

**Table 2:** ***Cryptosporidium *** **infection rates among dogs of different ages**

**Age**	**No. samples**	**No. positive**	**Prevalence (95% CI) (%)**
< 90 days	251	20	8.0 ± 0.06
90–180 days	150	4	2.7 ± 0.21
181–360 days	98	2	2.0 ± 0.28
> 360 days	271	3	1.1 ± 0.08
Total	770	29	3.8 ± 0.05

All 29 *Cryptosporidium*-positive samples were successfully amplified with nested PCR at the SSU rRNA locus. RFLP analysis of the SSU rRNA gene products revealed that these samples belonged to *C. canis*. DNA sequencing of the SSU rRNA PCR products from 12 *C. canis*-positive samples confirmed the identity of the species, with 100% similarity to dog- and human-derived *C. canis* sequences in GenBank [5]. To further evaluate the molecular characteristics of *C. canis* in this study, two *C. canis* isolates were sequenced at the HSP70 locus and eight at the actin locus. As expected, the two HSP70 sequences were identical to those obtained previously from dog-derived *C. canis* (AF221529) and coyote-derived *C. canis* (AY120920). Similarly, the four actin sequences (EU754834–EU754837) were identical to that of a dog-derived isolate (AF382340). The other four actin sequences (EU754838–EU754841) were identical to each other, and differed from isolates EU754834–EU754837 and AF382340 by only one nucleotide. Phylogenetic analysis of the SSU rRNA, HSP70, and actin genes confirmed the identity of *C. canis* in this study.

As in this study, *C. canis* has been identified as the most common *Cryptosporidium* species in dogs around the world, although a few cases of *C. parvum*, *C. muris*, and *C. meleagridis* infection have also been reported [1,11,12,14,17,22,30,33-35]. Unlike *C. parvum*, *C. canis* appears to have a relatively high level of host adaptability. A few cases of *C. canis* have also been reported in both immunocompromised and immunocompetent individuals in recent years [15,36-40]. Although a recent study suggested that cryptosporidiosis from pet dogs and cats poses only a minimal zoonotic risk, the possible public-health implications of *C. canis* and the economic significance of *C. canis* infections in dogs and other animals cannot be ignored [41].

## Conclusion

In the present study, we described the occurrence and molecular characteristics of *Cryptosporidium* in dogs in China using a large number of samples. This provides useful information that may extend our understanding of the prevalence of *Cryptosporidium* infections in dogs. China has a large number of dogs, all of whom are in close contact with humans. Therefore, because only *C. canis* was identified in dogs in this study, it is possible that *C. canis* is an emerging zoonotic infectious agent in some parts of China.

## Methods

### Ethics statement

Before beginning work on the present study, we contacted animal owners and obtained their permission to include their animals in the study. All animal experiments were conducted in accordance with the Chinese Laboratory Animal Administration Act 1988. Before the experiment, the protocol of the study was reviewed and approved by the Research Ethics Committee of Henan Agricultural University.

### Fecal samples and microscopic examination

Fresh fecal samples were collected from March 2006 to December 2009 from 26 locations in different places in Henan Province, China, including kennels, pet shops, pet hospitals, and a police canine center. A total of 770 samples were used in the study, including those from animals aged < 90, 90–180, 181–360, and > 360 days (Table 2). Fecal samples of at least 25 g were examined for the presence of *Cryptosporidium* oocysts using bright-field microscopy of the fecal material that had been concentrated with the formalin–ethyl acetate sedimentation method and Sheather’s sugar flotation technique [24]. *Cryptosporidium*-positive samples were stored in 2.5% potassium dichromate at 4°C.

### DNA extraction

The genomic DNA from nine *Cryptosporidium*-positive samples from kennels, identified between 2006 and 2007, was extracted from the purified oocysts by discontinuous density sucrose gradient centrifugation using a MagExtractor–Genome kit (Toyobo Co., Ltd., Osaka, Japan). The genomic DNA from the remaining 20 *Cryptosporidium*-positive samples from kennels, pet shops, and pet hospitals, identified between 2008 and 2009, was isolated using an E.Z.N.A.® Stool DNA Kit (OMEGA Biotek Inc., Norcross, GA, USA). DNA samples were stored at –20°C before molecular analysis.

### Cryptosporidium genotyping

The SSU rRNA gene of *Cryptosporidium* was amplified from microscopy-positive samples using nested PCR, according to previously described procedures, and restriction fragment length polymorphism (RFLP) analysis [25], using the restriction enzymes *Ssp*I and *Vsp*I (Fermentas, Shenzhen, China). Two-directional sequencing of 12 representative samples (two from pet hospitals, four from pet shops, and six from kennels) was performed with an ABI Prism 3730 DNA Analyzer (Applied Biosystems, Foster City, CA, USA) using the Big Dye Terminator v3.1 Cycle Sequencing Kit (Applied Biosystems). Some of the positive samples were further characterized by sequence analyses of the HSP70 gene (the two from pet shops and another two from kennels) and actin gene (the two from pet hospitals, the two from pet shops, and another four from kennels). The identities based on the gene loci of these samples were similar to those of the sequenced SSU rRNA samples. The primers and procedures used for these two loci were taken from previous studies [26,27]. The sequences of the actin genes were analyzed after the genes were cloned into the pGEM1-T Easy vector (Promega, Shanghai, China).

### Data analysis

The sequences were analyzed using ClustalX 1.83 (http://www.clustal.org/) and Phylip 3.69 (http://cmgm.stanford.edu/phylip/). Representative sequences of the *C. canis* isolates were deposited in GenBank under accession numbers EU754826–EU754833 (SSU rRNA), EU754834–EU754841 (actin), and EU754842–EU754843 (HSP70). The *χ*^2^ test was used to compare the *Cryptosporidium* infection rates in the different groups of dogs. Differences were considered significant at *P* < 0.05.

## Competing interests

The authors declare that they have no competing interests.

## Authors’ contributions

LXZ conceived and designed the experiments; FCJ, MQ, and HPD performed the experiments; FCJ and RJW analyzed the data; XYH and CSN contributed reagents/materials/analysis tools; FCJ and LXZ wrote the paper. All authors have read and approved the final version of the manuscript.
